# Exploring medication self-management in polypharmacy: a qualitative systematic review of patients and healthcare providers perspectives

**DOI:** 10.3389/fphar.2024.1426777

**Published:** 2024-09-13

**Authors:** Ran Jin, Caiyan Liu, Jinghao Chen, Mengjiao Cui, Bo Xu, Ping Yuan, Lu Chen

**Affiliations:** ^1^ School of Nursing, Nanjing Medical University, Nanjing, China; ^2^ School of Nursing, Nanjing University of Chinese Medicine, Nanjing, China; ^3^ Nanjing Drum Tower Hospital, The Affiliated Hospital of Nanjing University Medical School, Nanjing, China

**Keywords:** polypharmacy, self-management, health system, qualitative, systematic review

## Abstract

**Purpose:** Polypharmacy presents many challenges to patient medication self-management. This study aims to explore the self-management processes of medication in polypharmacy from the perspectives of both patients and healthcare providers, which can help identify barriers and facilitators to effective management.

**Methods:** A systematic review of qualitative studies was performed by searching seven databases: PubMed, Web of Science, Cochrane Library, Embase, CINAHL, PsycINFO, and MEDLINE, from their establishment until August 2024. The Critical Appraisal Skills Programme (CASP) tool was employed to evaluate the quality of the studies included. The extracted data were then analysed thematically and integrated into The Taxonomy of Everyday Self-management Strategies (TEDSS) framework.

**Results:** A total of 16 studies were included, involving 403 patients and 119 healthcare providers. Patient management measures were mapped into TEDSS framework, including categories such as medical management, support-oriented domains, and emotional and role management.

**Conclusion:** Enhancing patients’ proactive health awareness, improving medication literacy, balancing lifestyle adjustments with medication therapy, dynamically reviewing and optimizing medications, strengthening patients’ social support networks, and helping patients integrate medication management into their daily life are the key elements that can effectively assist patients in self-managing their medications. Future interventions to improve patient medication self-management ability should be designed for these issues.

**Systematic Review Registration:** https://www.crd.york.ac.uk/PROSPERO/, identifier CRD42024524742.

## 1 Introduction

Polypharmacy, often defined as the use of multiple medications, has become increasingly prevalent worldwide and is now a significant public health concern (Donaldson et al., 2017). Factors such as the aging population, the rising burden of chronic diseases, and advancements in medical technology and diagnostic capabilities have led to a growing reliance on multiple medications in daily treatment regimens (elara et al., 2022). Globally, the prevalence of polypharmacy in the general population is approximately 37%, with higher rates observed in older individuals at 45% (Kim et al., 2024). Though polypharmacy is often defined as the use of five or more medications (Varghese et al., 2024; Nicholson et al., 2024), there is no consensus on its exact definition (Masnoon et al., 2017). The World Health Organization emphasizes that beyond numerical definitions, the focus should be on evidence-based practices to reduce inappropriate polypharmacy (Varghese et al., 2024).

Appropriate polypharmacy is crucial for managing complex health conditions, but inappropriate polypharmacy, characterized by the use of unnecessary or potentially harmful medications, can lead to significant adverse outcomes (Hoel et al., 2021). As the number of medications used increases, the risk of drug-related problems grows almost exponentially, including drug-drug and drug-disease interactions, adverse drug reactions, and potentially inappropriate medications (Wastesson et al., 2018). Healthcare systems often lack shared records, leading to patients receiving duplicate or interacting prescriptions from multiple providers, and sometimes additional medications to treat adverse reactions caused by other medications (Wang X. et al., 2023). The use of multiple medications also increases the risk of adverse drug events, such as falls (Roitto et al., 2023), weakness (Palmer et al., 2019), cognitive, physical, and emotional dysfunctions (Khezrian et al., 2019), and even rehospitalization (Prasad et al., 2024) and death (Chang et al., 2020), imposing a significant cost burden on healthcare systems (Hoel et al., 2021). Besides, The increase in the number of medications is associated with low medication management ability (Wastesson et al., 2018). The complexity of managing multiple medications, especially with different dosing schedules or special storage conditions (Albert et al., 2022), can lead to reduced medication literacy (Wang W. et al., 2023) and difficulty in self-management. The high economic cost of medications can be a barrier, particularly for those without adequate insurance coverage (Holbrook et al., 2021). The psychological stress from side effects or fear of interactions, as well as social pressures such as disrupted social schedules and social stigma, can also lead to patients skipping doses or stopping medication (Widyakusuma et al., 2023), resulting in incorrect usage, affecting the effectiveness of treatment.

Medication self-management is a complex and crucial process that involves a range of services aimed at improving clinical outcomes. These services include completing medication reviews and health assessments, monitoring treatment plans and the effectiveness and safety of therapies, as well as providing education and promoting self-management. This process goes beyond simple medication adherence (Cadel et al., 2021). Self-management encompasses three domains: medical, emotional, and role management (Lorig and Holman, 2003). Through extensive conceptual reviews and interviews with patients suffering from neurological disorders, Audulv et al. (Audulv et al., 2019) developed the Taxonomy of Everyday Self-management Strategies (TEDSS) framework. This framework aims to provide a structured understanding of the strategies patients use to manage their health in daily life. The TEDSS framework consists of five goal-oriented domains (internal, social interaction, activities, health behavior, and disease controlling) and two additional support-oriented domains (process and resource). These domains correspond to the traditional concepts of medical, emotional, and role management in self-management.

To meet a broader range of self-management needs, Cadel et al. (Cadel et al., 2020) refined the TEDSS framework based on analyses of attitudes and experiences of medication self-management among patients with spinal cord injuries and healthcare providers. These adjustments categorized the framework into medical management (disease controlling strategies and health behavior strategies), support-oriented domains (process strategies and resource strategies), and emotional and role management (activities strategies, internal strategies, and social interaction strategies). These refinements allow researchers to more comprehensively capture the diverse methods patients use to manage their medications in daily life. For instance, disease controlling strategies and health behavior strategies help patients effectively manage chronic conditions. Process strategies and resource strategies focus on how patients obtain and utilize necessary resources and implement these strategies. Additionally, activities strategies, internal strategies, and social interaction strategies address how patients fulfill their social roles, manage emotions, and engage in social interactions in their daily lives. This comprehensive framework helps to reveal the actual challenges and needs in patients medication self-management, providing healthcare providers with valuable insights to offer more targeted and supportive care.

In recent years, the phenomenon of polypharmacy has garnered widespread attention, and some qualitative studies on medication self-management have been conducted. However, the results of single qualitative studies can not comprehensively and accurately reflect the medication self-management in polypharmacy. Although some studies have synthesized the medication self-management experiences of polypharmacy among patients, these results tend to focus more on medical management, less on other aspects of self-management, and lacking the perspective of healthcare providers (Eriksen et al., 2020). As medication self-management research evolves, reviews need to be updated to better guide clinical practice. Therefore, the purpose of this systematic review is to utilize the TEDSS framework to gain an in-depth understanding of the barriers and facilitators influencing medication self-management, considering the perspectives of both patients and healthcare providers. The results of this comprehensive study may provide valuable information for designing and effectively implementing medication self-management interventions for patients with polypharmacy, potentially improving patients quality of life and reducing the burden of medication.

## 2 Methods

### 2.1 Study design

We adopted a systematic review as it allows for an in-depth understanding of multiple study outcomes, facilitates the formation of novel theoretical or conceptual models, and provides substantiation for the creation, implementation, and evaluation of health interventions (Tong et al., 2012). This review was performed following Preferred Reporting Items for Systematic Reviews and Meta-Analysis (PRISMA) 2020 guidelines and was registered in the International Prospective Register of Systematic Reviews (PROSPERO), with the registration number CRD42024524742.

### 2.2 Search strategy

The search was carried out across seven databases, including PubMed, Web of Science, Cochrane Library, Embase, CINAHL, PsycINFO, and MEDLINE. The search period ranged from the establishment of each database to August 2024. Based on our research objectives, we identified relevant search terms for three key concepts: polypharmacy, self-management, and qualitative research. We used a search strategy that combined medical subject headings (MeSH) and free-text terms, with adaptations tailored to the characteristics of each database. The detailed search strategy for the databases is provided in Supplementary Appendix S1.

### 2.3 Inclusion and exclusion criteria

Following the principles of the SPIDER tool (Sample, Phenomenon of Interest, Design, Evaluation, Research type), we devised a sensitive and comprehensive search strategy (Methley et al., 2014). The samples included patients with multimorbidity and polypharmacy, as well as healthcare providers; Studies that focused solely on patient experiences or solely on healthcare provider experiences were also considered. We defined multimorbidity as the coexistence of two or more chronic diseases and polypharmacy as the use of five or more medications. The phenomenon of interest was the attitudes and experiences of patients who manage their medication regimens within the context of daily life and healthcare providers who implement interventions to promote patient medication self-management; The study design included ethnography, grounded theory, phenomenology, or narrative research; The evaluation consisted of patients’ and healthcare providers’ attitudes and experiences towards medication self-management; and the type of study was qualitative.

Studies were excluded if (1) patients were in the terminal stage of illness and receiving palliative care or had cognitive impairment, as they were unlikely to reflect the wider population’s attitudes and experiences of medication self-management in daily life; (2) the type of study was reviews, case studies, editorials, conference abstracts, commentaries, or research protocols; (3) the full text of the study was not available; or (4) English was not the language of publication for the original research report.

### 2.4 Study selection

**FIGURE 1:**
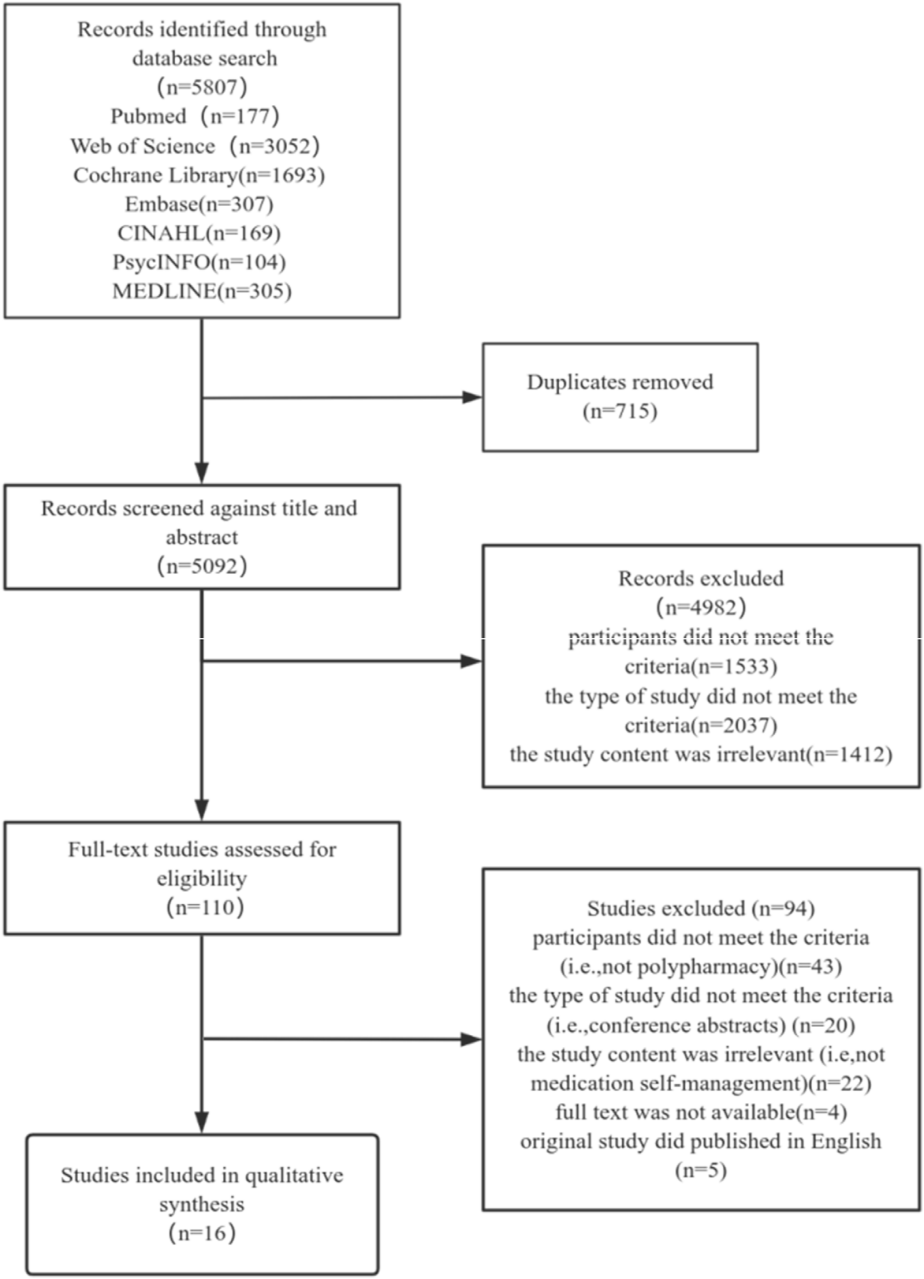
PRISMA flow diagram of the literature screening.

The studies retrieved in this research were imported into Endnote X9, and duplicate articles were removed. Two authors (RJ and CYL) independently screened the titles, abstracts, and full texts based on the inclusion and exclusion criteria to obtain the final included studies. Any disagreement was evaluated by another author (LC) and discussed to reach a consensus. The detailed flowchart is illustrated in Figure 1.

### 2.5 Data extraction and synthesis

Two authors (RJ and CYL) independently evaluated and extracted key data from each eligible study, including information on authors, year of publication, country, study aim, participants, number of medications, data collection and analysis methods, conceptual or theoretical framework, and main findings. The findings were used in a deductive process into the TEDSS framework, which is the framework thematic synthesis approach (Brunton et al., 2020). Participant citations from 16 eligible studies were imported into NVivo 14 software. These citations were coded by two authors (RJ and CYL) independently to develop a mutual understanding of the coding framework and themes. Any disagreements or uncertainties were evaluated by the full research team and discussed to reach a consensus.

### 2.6 Quality appraisal

This study assessed the methodological quality of the included studies using the Critical Appraisal Skills Programme (CASP) qualitative checklist (Eriksen et al., 2020). Two authors (RJ and CYL) independently conducted the assessment, and no studies were excluded based on methodological quality. Besides, the same two authors independently used the ‘Confidence in the Evidence from Reviews of Qualitative research’ (GRADE-CERQual) approach (Lewin et al., 2015) to assess the confidence in each finding. Disagreements were discussed and resolved with another author (LC) to reach a consensus.

### 2.7 Theoretical framework

As previously described, the findings of this study are related to the TEDSS framework (Cadel et al., 2020). The TEDSS framework, originally created by Audulv et al. (Audulv et al., 2019) for more general self-management, was adapted to comprehensively capture the diverse methods patients use to manage their medications in daily life and to reveal the actual challenges and needs in medication self-management. The improved TEDSS framework includes three domains: medical management (disease controlling strategies and health behavior strategies), support-oriented domains (process strategies and resource strategies), and emotion and role management (activities strategies, internal strategies, and social interaction strategies). In the Results section, we provide detailed explanations for each topic.

### 2.8 Rigour, trustworthiness, and reflexivity

We analysed quotes from participants to provide detailed explanations of the topic. Our team, consisting of academic nurses, research assistants, and clinical experts trained in qualitative methods, spans different academic career stages and cultural backgrounds. This diversity significantly aids in reducing personal biases during the processes of literature screening, quality assessing, and result interpreting. Additionally, to address and resolve any disagreements, the team held regular meetings throughout the study. The authors (CYL, JHC, MJC, BX, PY, and LC) have extensive research experience and have previously published systematic reviews. BX, LC, and PY are clinical experts with rich clinical practice experience in promoting patient medication self-management. This review, part of a master’s thesis, explored the barriers and facilitators to medication self-management in patients with polypharmacy, providing theoretical support and practical guidance for the development of interventions. Additionally, seven participants, including four patients and three healthcare providers (HCPs), reviewed the integrated themes and their recommendations were included in the determination of the final themes.

## 3 Results

### 3.1 Search results and study characteristics

A total of 5,807 records were identified. After removing duplicates and screening the titles and abstracts, 5,697 studies were excluded. At full text assessment, 16 studies were included. The study flowchart is shown in Figure 1.

Five studies were conducted in the United States, two studies each in the United Kingdom, Australia, and Canada, and one study each in Sweden, Germany, Ireland, Thailand, and Netherlands. A total of 403 patients and 119 HCPs were included. The data collection method was mainly interviews, twelve studies used interviews, one study combined interviews with observations, while two studies used focus groups and one study used narrative inquiry. Methodological analysis was mainly thematic analysis, six studies used this method, while four studies used constant comparison method, three studies used content analysis, two studies used framework analysis and one used hermeneutical analysis and content analysis. Table 1 shows the specific details of the 16 studies included.

**TABLE 1:** Study characteristics.

Author/ (year) Country	Study aim	Participants	Medication numbers	Data collection method (DC) and analysis (DA)	Conceptual/Theoretical framework	Main findings
(Holmqvist et al., 2019) Sweden	explore and describe older persons’ experiences of evaluation of their medication treatment	20 community‐dwelling older persons (age 75–91 years)	mean (range): 12.7 (6‐26)	DC: semi‐structured interviews DA: inductive qualitative content analysis	the medication use model	Theme 1: Own role in the evaluation Theme 2: Views of evaluation received
(Fried et al., 2008) United States	examine the ways in which older persons with multiple conditions think about potentially competing outcomes in order to gain insight into how processes to elicit values regarding these outcomes can be grounded in the patient’s perspective	66 participants aged 65 years and older	median (range): 7 (5-14)	DC: focus groups DA: constant comparison method	NA	Theme 1: Recognition of competing outcomes Theme 2: Understanding of the likelihood of outcomes Theme 3: Disease-specific versus global outcomes
(Schöpf et al., 2018) Germany	explore elderly patients’ and general practitioners’ (GPs’) perceptions of communication about polypharmacy, medication safety and approaches for empowerment	6 patients at least 65 years old with polypharmacy; 3 GPs (general practitioners)	mean ± SD: 8.2 ± 2.6	DC: semi-structured interviews DA: a framework analytical approach	NA	Theme 1: differing medication plans: causes? Theme 2: dialogue concerning medication: whose responsibility? Theme 3: supporting patients’ engagement: how?
(Tudball et al., 2015) Australia	how consumers residing in Australia experience and manage their multiple medicines while travelling	35 community dwelling participants, most aged over 50 years	range: 5-25	DC: face-to-face, narrative interviews DA: constant comparative method	NA	Theme 1: Planning for the trip Theme 2: Organising and packing medicines for the trip Theme 3: Maintaining usual routines while travelling Theme 4: Travelling overseas
(Williams et al., 2005) United Kingdom	explore attitudes and practices to medication regimens among patients already in receipt of multiple medications, and to assess whether a combined tablet would be perceived as advantageous	92 men and women aged >40 years	range: ≥6	DC: focus groups DA: a framework analytical approach	NA	Theme 1: Daily drug routines Theme 2: Problems with regimens Theme 3: Attitudes to a combined pill
(Hannum et al., 2021) United States	understand clinical team members’ perceived barriers to medication safety in preparing older patients to return home and to identify potential redesign strategies that reduce ADEs throughout the transition	37 clinical team members representing 10 different professional roles involved in providing transitional care	NA	DC: semi-structured interviews DA: thematic analyses	Systems Engineering Initiative for Patient Safety (SEIPS) 2.0 framework	Theme 1: Streamlining and coordinating clinical management of medication regimens across care settings to better prepare patients for the transition to home Theme 2: Building patient capacity and engagement in self-managing medications at home Theme 3: Redesigning the transitional process to be more patient centered
(Cossart et al., 2022) Australia	examine medication-taking behaviors of kidney transplant recipients transplanted at 60 years of age or older	14 older adult kidney transplant recipients	median (min–max): 13 (10–26)	DC: semi-structured interviews DA: thematic analyses	The Theory of Planned Behaviour	Theme 1: Perceived Health Literacy Toward Medicines Theme 2: Support Networks Theme 3: Adjusting Health Expectations Theme 4: Motivators for Self-Care Theme 5: Medication Management Theme 6: Approaches to Medication Taking
(Previdoli et al., 2024) United Kingdom	examine in depth how older people with mild to moderate frailty manage their polypharmacy regimens at home	32 patients aged 65 years or older with mild or moderate frailty and taking five or more medicines	range: 5-15	DC: semi-structured interviews DA: reflexive thematic analysis	resilient healthcare framework	Theme 1: Managing many medicines is a skilled job I did not apply for Theme 2: Medicines keep me going, but what happened to my life? Theme 3: Managing many medicines in an unclear system Theme 4: the support with medicines I value and that makes my work easier Theme 5: My medicines are very familiar to me. There is nothing else I need (or want) to know or worry about
(Foley et al., 2022) Ireland	explore the experience of self-managing multimorbidity among older adults, with a focus on medication adherence	16 people with complex multimorbidity aged 65 years or older	mean (range): 13.2 (9-18)	DC: individual semi‐structured interviews DA: reflexive thematic analysis	NA	Theme 1: Amplified burden Theme 2: Pathways towards relief
(Guilcher et al., 2019) Canada	explore healthcare providers’ conceptualization of factors impacting medication adherence for persons with SCI/D	32 healthcare providers with varying clinical expertise	NA	DC: individual semi‐structured telephone interviews DA: constant comparative	ecological model of medication adherence	Theme 1: Micro-level factors Theme 2: Meso-level factors Theme 3: Macro-level factors
(Vatcharavongvan and Puttawanchai, 2022) Thailand	explore how older patients with polypharmacy manage medications at home in a primary care unit (PCU)	19 patients (mean age = 69 years)	median (range): 6 (5-10)	DC: in-depth semi-structured interviews and observations DA: thematic content analysis	NA	Theme 1: Medication storage system Theme 2: Factors affecting medication adherence
(Hernandez, 2017) United States	explore the personalized meanings study participants ascribed to the experience of managing polypharmacy in practice	15 NPs (nurse practitioners) with self-identified as caring for geriatric patients taking multiple medications	NA	DC: narrative inquiry DA: thematic analysis	The metaphorical three-dimensional narrative inquiry space	Theme 1: Mastering the art of the puzzle Theme 2: It takes a village Theme 3: Power in knowledge
(Dijkstra et al., 2022) Netherlands	explore how older people living at home self-manage their medication and what considerations and decisions underpin their medication self-management behaviour	60 patients with a median age of 86.5 (IQR 78–89)	median (IQR): 8 (6-11)	DC: semi-structured interviews DA: content analysis with a directed approach	three phases of medication self-management (initiation execution, and discontinuation)	Theme 1: The initiation phase Theme 2: The execution phase Theme 3: The discontinuation phase
(Vandermause et al., 2016) United States	examine the experiences of older adults with multiple chronic medical conditions when a new medication was added to their existing multiple medication regimen	15 patients aged 60 years and older, 5 or more medications plus a new prescription and 3 chronic medical diagnoses	mean ± SD: 11.9 ± 4.4	DC: In-depth hermeneutic interviews DA: hermeneutical analysis and content analysis	NA	Theme 1: Preserving Self: Living With Chronic Conditions and Being With Healthcare Providers Theme 2: Engaging Providers in Visioning Health
(Guilcher et al., 2020) Canada	explore the experiences of healthcare and service providers supporting medication therapy management	32 healthcare and service providers	NA	DC: semi-structured interviews DA: constant comparative analysis	NA	Theme 1: Professional contribution to medication therapy management Theme 2: medication therapy management barriers and enablers
(Jallow et al., 2024) United States	explore medication safety strategies used by community dwelling older adults aged 65 years and older who took five or more prescription medications	9 residents from the retirement community and 19 patients from 2 clinics with a median age of 75	mean ± SD:7.7 ± 2.5	DC: semi-structured interviews DA: deductive thematic analysis	Systems Engineering Initiative for Patient Safety (SEIPS) 2.0 model	Theme 1: Collaborating With Prescribers Theme 2: Collaborating With Pharmacists Theme 3: Learning About Medications Theme 4: Safe Practices at Home

### 3.2 Quality of studies included

The CASP tool assessment indicates that most of these studies (n = 12) demonstrate minor to moderate methodological limitations. These studies articulate clear objectives and employ qualitative methodologies appropriately and all of the studies put a clear statement of findings and were of value. While these studies except one overlooked the relationship between researcher and participants. Additional information can be found in Supplementary Appendix S2.

### 3.3 Confidence in review findings

The GRADE-CERQual approach assessment indicates that the majority of the finding statements demonstrate moderate or high confidence. Additional information can be found in Supplementary Appendix S3.

### 3.4 Findings

From the 16 studies included, we extracted 320 citations and categorized them using the TEDSS model to describe the self-management of medication. Following and Table 2 are some of the key exemplary citations.

**TABLE 2:** Key supporting quotes.

Theme	Subtheme	Supporting quotes
Medical Management	Disease Controlling Strategies	“I have an idea of what each shape is. I knowm water pill is a little, pink pill. My blood pressure, is a long blue pill.”
		“I do not have a printed medication list, but I have photos of the plastic pouches [of the MDD system] in my mobile phone. I always have my mobile phone and thus information of my medications. When I go to a physician, I show him the photo.”
		“I fit something else that I can take that the insurance would cover, and it’s the same medicine, maybe a generic or something, then I will see if they will prescribe that one instead of the one I have to pay out of pocket for that’s more than what I can afford.”
		“If they’re not getting their refills or if they’re not coming back on a regular basis, that’s something that you do have to monitor or that’s something that would signify that [the medication is] not working.” (HCP)
		“So there are high-risk medications that we as case managers go. We need to make sure everything’s onboard with this, that they’re getting what they need to be done, like the levels done. Are they doing what they’re supposed to be doing to make sure that the medication’s effective for them? make sure that they’re going to a clinic to get their blood tested. ” (HCP)
		“I do not know where I can find an expiratory date, but I normally finish the remaining medications before taking newly received medications. I do not want to waste the medications.”
		“ [I kept medications] in a drawer. [Some] are hung on a bed because I believe the room is not hot and moist. Some medications should not be kept in a hot and moist room, should they? I organize them all myself. A doctor told me to keep them in a dry and cool location. ”
		“Well, I suppose you need a few spatial skills to make sure you put them [medicines] in the right [compartment of the compliance aid], I mean and you need to understand the ones that are morning, and the ones that are evening. And I think you need to concentrate as well. ”
		“So I always check the colors of the pills before I take the pills. I use eight pills, six white pills, a pink one and a yellow one. But that pill, the color has been changed, the pill was yellow but now it is white. I get confused because my system tells me to take six white pills each day.”
		“... sometimes I fall asleep during the day. So, I have medications for 12 o’clock, but when I sleep at that time and wake up at let’s say 12.30 o’clock, it is too late to take it. Someone told me to not take the medications when the intake moment has passed, so I do not take the pills.”
		“ [For] traumatic accidents, spinal cord injury and brain injury can go hand-in-hand [individuals with brain injuries sometimes have] difficulty remembering to take their medications [and might be] less likely to take their medications … forget to take [their medications or] think it does not matter if they take [their medications].” (HCP)
		“Well, they have said that it is good if you can stay under ten [blood sugar] One day we had nineteen and I understand that it is not good.”
		“I have been trying to convince my doctor that I do not need the cholesterol medication any longer, because it has zapped me of my strength, and it is debilitating.”
		“Because I know that when I got blood pressure medications then, then the doctor said ‘If there is anything that you feel then, that you have not felt before, because you are taking this medication, you will have to let us know’ But I have never felt that.”
		“My neighbor has diabetes. Once his medications ran out. He asked me for medications. I had plenty of them, so I gave him ten packages approximately 100 pills. He was delighted and thanked me for the medication.”
		“I throw it in the garbage bin. The medications will be disposed of in the garbage bin in the pharmacy, so I may do that at home as well.”
	Health Behavior Strategies	“... so I’ve noticed that there is one medication, and I forgot the name, but it is one that they have to take and they’re not able to eat their meal after. And one of the things that impacts them taking that one on a regular basis is that they have to wait a certain amount of time before they’re able to eat and I think it’s like 2 hours or so. So, I guess the activities that they have to conform to, or the conditions while taking their medication.” (HCP)
		“I worked hard on my diet-kept track.”
		“I would like to be a bit more active. I would like to get this all over, and feel this is not a burden anymore, I’d like to get back to my routine. I used to get up every morning and walk on a treadmill. I used to clean up the backyard.”
Support-Oriented Domains	Process Strategies	“But he did not say anything last Friday. So it is happy and pleased. It must be.”
		“... whether they actually see a benefit or not and sometimes with some medications, it’s clear that you see benefits. For instance, if you take your medications for spasticity, then you really see it. But sometimes if you actually prescribe something that they do not necessarily see the direct benefits, then it’s hard for them to be compliant unless they really understand what that’s for.” (HCP)
		“Expectations are a big thing. Specifically, like if they are taking something for a medication like a pain medication where you can see the results or you’re expecting a change and you are able to notice that, it’s a lot different than if you’re treating like hypertension where, you know, they cannot really see the results and so, the expectations of what they are going to get out of the medication.” (HCP)
		“More medicines means older and a declining condition, whether or not it’s true.”
		“I want to get off, reduce the Xanax that I’m taking, but that’s for the stress and everything that I’ve just been through. So I have not done that because I’ve tried like breaking the pill in half, and my stomach is just rolling. So i take the other half and it settles down. But eventually, I will get off of it.”
		“I just did nae like taking them cos in case I had side effects or that but I would nae take tablets, aspirin or anything. I would rather go about with a sore head until it cleared itself but nowadays. (laughing)”
		“Well for the amount I have to take, I do not know about these guys, but I take quite an amount of tablets, and do they not fight against one another? I mean is there not chemicals in one that’s going to be fighting against another?”
		“... they want to know more and they want to be educated more, but sometimes where they get their education, their resources are not, are not appropriate sites.” (HCP)
		“So, if you do not know why you are taking something, it’s very difficult to think, ‘okay, well I need to take this.” (HCP)
		“... do not usually include patients with spinal cord injuries. So, it’s hard to know if there needs to be any adjustments made for them because the data is not there.” (HCP)
		“... you know, recognizing that for the average primary care practitioner, they are going to have a very small number of these people and so, to expect that they, by themselves, can maintain a level of clinical expertise necessary or appropriate to the complexity or the specifics of the type of health problems and medication issues that spinal cord patient experience, I think that’s not reasonable.” (HCP)
		“... because I’m not like primarily focused on the SCI patients, I do not know that much about specifics that should be addressed. So, I probably do not provide education that might be more specific or tailored to them just because I’m unaware of that information and where to find it.” (HCP)
		“Yeah, it was overwhelming. And it was like in one ear and out the other. I could not concentrate on what they [doctors] were saying.”
		“I can’t read the text, the letters are too small. My daughter in law reads aloud the leaflets for me.”
		“I tell the doctor that my back hurts. He says, okay, take this. I say, what’s it for? He says, it helps with the pain. I say, okay, thank you. I take the medicine [ask no further questions].”
		“They’ll send an information sheet, and so I read that very thoroughly. If I have any questions about it, I’ll ask my doctor when I see him.”
		“I think it’s probably easier taking the lot together. Yeah. Because if you were taking them in ones I’d say you’d be forgetting them half the time. you’d be missing them.”
		“... the [medication] for the thyroid. This one I would like to leave out. The daughter has said: Just do not take them. Then I said: I know, I have already taken that for years. And leaving them out, I do not know. I will talk to the GP, that at least one or two.”
		“I made the decision to stop taking statin because I feel better. Living is more important. if you said to me, right you can have pain free now for the next year and a really nice time and then you will die, I would say, I’ll take it.”
		“I had terrible trouble because if you take your tablets at 8 o’clock in the morning, at 8 o’clock at night you do not know where you’re going to be anyway, and I’d be in bed and I’d think, oh, forgot my tablet. Soit was the chemist actually, who was going through them, and he said: You can have one that you just take once a day. So, that makes that a lot easier for me, because it’s just once a day.”
		“I think number one [for medication adherence] would be like the frequency. So, I think people can take things once a day, but I think you are really pushing it when you are asking them to take things twice and three times a day. The other thing that I find is like the actual volume of pills. So, when I can I will also look for opportunities to combine medications so that there would be less pill burden.” (HCP)
		“The longer the person has [the] injury, the more insight they have into their body and you have to give credit. I could say once they are, you know, more medically stable, you really need to listen to them and listen to how they want to live their life and how you can complement their life with the medication to control the spasms, to control the pain at the right time so that they can have a meaningful life.” (HCP)
	Resource Strategies	“I know nothing about this. I mean, you have to bow to the knowledge that the doctor has. You got to believe that, in some way, it helps.”
		“No, but I have a little in me that they know what to do. I trust them.”
		“I remember all medications because I take them every day. I know which medications have to be taken before a meal in the morning, and I take them before breakfast.”
		“We’re navigators in trying to help them to keep their things in order, but we do not want to do it for them. We want to make them as independent as possible. So I’ll sit there and say, Okay, now, take the paper he gave you and okay, he made that change. Now take that pill and put it in a box and let me see you. Because we want them to do it and not us do it for them.” (HCP)
		“... like to be involved in the decision making and I think if you involve them, they are actually probably more likely to accept the therapy and be adherent.” (HCP)
		“I think it’s important to understand what the medications are for, and you know, things, things that they could cause. And I just find it interesting.”
		“My vision and hearing are not that good. I cannot read labels. My niece writes instructions in big letters. Sometimes, she reads the labels. She tells me to take these medications after breakfast. I need her help. She does not organize them every day. Medications from PCU are put in baskets. one for morning and the other for the evening.”
		“I just think that the young ones, the children or whoever takes care of you, should influence you. I know that from my daughters, who say: Now you are going to the doctor and also tell him that and so on. Perhaps that is less the case with some people who are totally alone or so on. So, one sometimes needs a bit of a push. (laughing)”
		“... now that I got this plan from the doctor’s office on how they intended to do it. It is the first time they have reported what they have been thinking. And how they have planned to manage it.”
		“Yes, but it’s as different as night and day [to see the same physician instead of different ones] because then you can just pick up where you left off instead of having to go over everything from the beginning, everything that has happened and so on.”
		“... she said ‘You should take this pill’ and I had no idea what it was. She could have sat down [bedside] and said that this one is for this and that and so on, they do not have time for that.”
		“If you go and take blood tests, then you ought, at least, to get some kind of result, one would think. Because the doctor, he gets the test results, but I do not.”
		“ [I have not had a review] for the last 2 years, because you cannot see a GP. You cannot get to speak to anybody.”
		“If you feel put off by somebody, you’re not going to feel free to ask questions or ask them to do something for you. But the one, the pharmacy I’m going to now, they are very good about talking to me about my medicines and will answer any question that I might have.”
		“ [Caregivers] may get confused and give the wrong [medications].” (HCP)
		“It is so important for the patient’s family, or significant other, or significant friend, to be aware of the geriatric patient. Be aware and notice if the patient is unable to put medications in order or take them every day.” (HCP)
		“ [creating a] family atmosphere [with the patient and their caregivers to create] this background bond … [so they] do not feel scared to be honest with you.” (HCP)
		“With this population, if it’s the inability of them sometimes to maybe get into the pharmacy so that they are not getting the one-on-one counselling, so they can fall through the cracks. So, someone, you know, everybody just assumes it’s easier just to keep sending the medication [medication delivery] versus anyone going out having the discussion with them about it are big barriers [to supporting patients with SCI/D with their medications].” (HCP)
		“I rely on either the specialist provider and reach out to them if I’ve got questions or our local pharmacist who can also access other pharmacists, for instance, who might be in particular clinics where they are providing care to a lot of spinal cord patients.” (HCP)
		“... we have support through PCVC (personal computer video conferencing) support that they do not have to necessarily come in in-person or virtual visits.” (HCP)
		“You trust a patient who can accurately tell their medications more than one who reads it from a list and then the list is changed three times because of a doctor or another doctor or a nurse.” (HCP)
		“The time of the doctors, for example, patient has 10, 20 medications. The nurses, they do not have really time. You will see if you will audit not all patient is done the [medication reconciliation], or sometimes the patient does not know and the family does not know or they’re not a [hospital] patient, so they will not know.” (HCP)
Emotional and Role Management	Activities Strategies	“I place the pills beside the toothbrush. It reminds me every morning and evening to take the pills.”
		“So no matter where I go I’d always make sure that the tablets, they’d be the definite the one thing I would never go without are my medication.”
		“I will get it open and leave it open, and that’s the way I know I’ve taken it.”
		“And I cannot remember what it was, but something was going on and the [alarm] went off, and I thought, ‘I must take those tablets’ I think somebody was here. And so, I sort of said ‘Oh they’re going to go in a minute, I’ll take them as soon as they’re gone’ and, and they did not go!”
		“And you know, you carry your bag wherever you go. So you know if you sit down. if you go out and you have lunch out, you know that you’ve got to take your pill at lunchtime.”
		“You have to have, well I had little tricks to prompt me. It used to be feed the cat, take my warfarin, pour a glass of wine. see then, if there was a night when we did not have a wine, I’d forget my tablets.”
		“I find it’s the best way. So when I get them in the chemist they have them in, do you know these blister packs? So they’re all in the one. So I take them all then together.”
		“... well I had capsules, it’s not for high blood pressure it’s for acid and eh you know they’re in silver-backed and you’re to try and get the corner and pull them and I cannot, they’re annoying for me God knows what they’d be like for an 80-year-old.”
		“The last time I travelled, I actually ran out of medication, which was terrifying, I realised that I was about to run out of it about a week before I was going to run out. So I then worked out a routine, where I reduced the dosage to one-quarter of what I’d been taking and took that every day, instead of taking what I should have been taking.”
		“I went to Tamworth and was supposed to only be there for the day. The job went wrong and I was there for 3 days. I went to a doctor, he would not prescribe the medication. I went to the emergency room at the hospital. Because of the lithium, they then referred me to mental health and a psychiatrist came down. But it took about four and a half hours out of my day to actually go and do that.”
		“I’m going away for 8 weeks next year. So I’ve already checked online the websites for the embassies, consulates, whatever, for the different countries I’m going to, to find out what I can take in; what I need to have a letter from my doctor about; whether I need to have everything in the packaging it came in. I’m checking whether certain over the counter medications or alternatives are available in the countries that I’m going to, so that I do not have to travel with so much.”
	Internal Strategies	“I cannot stop taking these medications. Sugar and blood pressure go up and down all the time. Without medications, I cannot control them.”
		“I like it [organising and managing my medicines], I really like doing it, you know, it makes me feel good that I’m capable and I can do it and I know why.”
		“That becomes a problem particularly on a plane where you have to get up into the overhead lockers, get your bag out, get them out. People look at you, ask for a glass of water and take them and then it becomes awkward with the Novo-Pens. You roll the pen in your hand one way and roll it in the other way and then shake it up and down and then test it to see that it's coming out. You look like you’re taking a major drug lot. Then you put it in and do it. That is an effort in a plane.”
		“Well to be honest with you, if I had a choice I would prefer not to have to be taking the medication at all, do you know. But with my conditions, like, I do have no choice.”
	Social Interaction Strategies	“There’s no colour, race, anything. It’s just everybody’s a big family. It is, it really is. I’ve met some beautiful people.”
		“I’ve got another friend, whose daughter had a double lung transplant many years ago, so again, she understands completely what’s going on with me. If you’re talking to somebody who knows exactly what you’re talking about, it does make a difference.”

#### 3.4.1 Theme 1 medical management (77/320)

##### 3.4.1.1 Subtheme 1: disease controlling strategies (71/320)

In disease controlling strategies, close collaboration between patients and healthcare providers is essential to enhance patients’ ability to self-manage their medications and effectively control the disease in combination with non-pharmacological treatment. This involves the patient’s understanding and consent to use the medication, timely acquisition and replenishment of medication, careful review and verification of medication, proper storage of medication, correct medication usage, and continuous monitoring and managing the medication efficacy and side effects, as well as the appropriate handling of leftover medication.

##### 3.4.1.1.1 Understanding and consent to medication use

Patients typically obtain medication information through education from healthcare providers or medication lists, leading to their understanding and consent to use the medication. However, some patients may be reluctant to learn about the details of their medications, only remembering the shape and color, and relying entirely on healthcare providers for prescriptions. This can hinder their ability to participate in decision-making. Additionally, some patients may stop taking medication due to an overemphasis on side effects, which can reduce adherence. Therefore, healthcare providers should ensure that patients are fully informed about their medications to enhance their ability to manage them effectively.

“When I pick up the prescription, I’ll do like any normal person, I’ll read the instructions for taking the medication. When you get the medication, they tell you all about it. They give you a sheet.” (Jallow et al., 2024)

“Yeah no, well I do not even consider that [when asked about understanding each different medicine]. I’ve got to take them. That’s it. Full stop … You know, I have not got the knowledge about the pills so … so in other words, I’m trusting that they’re giving me the right stuff and I’ll go with that.” (Cossart et al., 2022)

“Miconazole (vaginal) cream, no I will not use it. I have read the patient information leaflet and I am scared of getting side effects.” (Dijkstra et al., 2022)

“I mean, sometimes they need all those medications, but they're understanding of what their medicines are and they're understanding of how to take them, why they take them, and what they're for, is really lost. They do not have good insight into what they're taking.” (HCP) (Hannum et al., 2021)

##### 3.4.1.1.2 Timely acquisition and replenishment of medication

Most patients recognize the importance of timely acquisition and replenishment of medication. Patients often use methods like taking photos of their medications or keeping personal records to provide a medication history. However, some may hide parts of their medication history (such as over-the-counter medications) or rely entirely on the healthcare system, which can lead to incomplete or inaccurate medication records, especially when interacting with different healthcare systems. Additionally, some patients prefer to use medications with lower out-of-pocket costs. Healthcare providers should assess patients’ medication acquisition practices to evaluate their management abilities, and consider the costs involved, such as transportation and time, and explore innovative ways to reduce these costs.

“One thing is you always have to kind of make sure you’re going to have your prescription and get it in on time and have your tablets, There’s that to taking all this medication.” (Foley et al., 2022)

“I do not bring a medication list with me to the physician, because all information about my medication is in their computer.” (Dijkstra et al., 2022)

“I would say with the elderly population they're on very limited incomes, so we have to be really mindful of that, and many times they'll come in here and we'll tweak their medications change it just a bit. Very confusing to an elderly person.” (HCP) (Hannum et al., 2021)

##### 3.4.1.1.3 Careful review and verification of medication

The dual communication between healthcare providers reviewing medications and patients verifying them is crucial for achieving optimal treatment outcomes and reducing inappropriate polypharmacy. However, some patients rely entirely on healthcare providers without verifying their medications, and may even take expired drugs. Some patients, having experienced medication errors, placed greater emphasis on verifying their medications.

“Our pharmacy technicians right now are assisting with getting appropriate medication lists. So sometimes at the discharge point, the provider realizes that the medication list is wrong, and they're trying to send out the right medication list, and so they want to make sure they have the old and the new to compare and write a good instruction sheet for the patient. Because if you do not tell them what to stop and you just tell them to start, they have some conflicting information. So our technicians help with that and they'll [also] help to get those medications filled at our outpatient pharmacy for them, and then bring them to the bedside. So they leave with the meds in hand.” (HCP) (Hannum et al., 2021)

“No, I do not check the medications [interviewer: ‘No, you do not check the correctness?’]. No, the pharmacy sends the medications and then well I assume that the medications are correct.” (Dijkstra et al., 2022)

“Once, I received the wrong medication by the pharmacy; since then, I always check the correctness.” (Dijkstra et al., 2022)

“I never take expiration dates into account, and I did not know that the dates are described on the medication packages.” (Dijkstra et al., 2022)

##### 3.4.1.1.4 Proper storage of medication

Medications that are not stored according to recommended temperature, light, and humidity conditions, or that are not properly kept in their original packaging, can have reduced therapeutic effectiveness. Many patients are unaware of the correct storage methods, or they prioritize convenience over proper storage, neglecting the necessary conditions. Additionally, healthcare providers often do not provide detailed instructions on proper storage. It is also crucial to remind patients to keep medications out of reach of pets and children.

“I do not know where I have to store the medications. I do not know anything about rules. I store all pills of my wife, so the MDD system (including furosemide, metoformine, sotalol, spironolactone, and enalapril maleate, [interviewer’s note]) and the other medications (tiotrus tiotropium, [interviewer’s note]), in the fridge. For me it is a logical place, so I store all the medications in the same place.” (Dijkstra et al., 2022)

“I keep medications in the bedroom and hang some on a bookshelf. Medications taken in the morning are kept in the kitchen both before and after a meal. There is a dining table there in the kitchen.” (Vatcharavongvan and Puttawanchai, 2022)

“My role is to take it regularly and [make sure] she [cat, does not] get into my medicine. I have to keep it out of sight.” (Jallow et al., 2024)

##### 3.4.1.1.5 Correct medication usage

Taking medications as prescribed is crucial for effective disease management. Healthcare providers note that some patients may intentionally or unintentionally not adhere to medication due to physical disabilities or cognitive misunderstandings. Additionally, some patients may be unsure about the correct way to take their medications, or what to do if they miss a dose. Others may self-administer over-the-counter medications to supplement their treatment without considering potential drug interactions, which can pose risks. Therefore, it is important to provide personalized assessment and guidance to these patients.

“I do not know the rules, I stir all pills in a glass of water and when they are mixed, I drink the water.” (Dijkstra et al., 2022)

“I did not know there could be interactions between my medications of my physicians and the OTC medications. I do not ask the pharmacist or the seller of OTC medications if it is safe for me to use it.” (Dijkstra et al., 2022)

“[I focus on improving] fine motor skills to pick up pills physically opening pill boxes.” (HCP) (Guilcher et al., 2019)

##### 3.4.1.1.6 Monitoring and managing medication efficacy and side effects

Patients with polypharmacy often suffer from several chronic conditions, necessitating close attention to continuous monitoring and managing medication efficacy and side effects. This allows for dynamic adjustments to medications based on the patient’s symptoms. It is important to note that some patients, particularly the elderly, may not recognize side effects, while others may have misconceptions about side effects, leading them to stop or alter their medications on their own. To address this, healthcare providers should inform patients in advance about potential side effects and encourage open communication to facilitate dynamic medication adjustments as needed.

“But they will keep track on me for five years now, with two blood samples every year. To see how it is then.” (Holmqvist et al., 2019)

“That is when, if you get side effects. Or you cannot notice it. That one has received ... too much? ... And one does not know. ... Side effects, it may be so different with that. Because you may feel slightly strange. It may be for other reasons. ... So, it’s not given that it’s the drugs either. That bothers me so.” (Holmqvist et al., 2019)

“If you have side effects, you do not have a choice. You just stop taking the medication because it could lead to something else happening.” (Fried et al., 2008)

“Well, often [the] side effects most people get are expected and a normal consequence, like if someone’s on gabapentin for neuropathic pain, and they feel some fatigue within the first few days, I will ask them - I will reassure them that that’s expected and it will likely improve. If it does not, then I’m willing to make a dose adjustment, change medications or stop that therapy altogether.” (HCP) (Guilcher et al., 2020)

##### 3.4.1.1.7 Appropriate handling leftover medication

It is important to address how patients store leftover medication. Some patients keep unused medications for future use but often neglect to check details such as expiration dates, which can lead to adverse outcomes. Additionally, some patients may share their medications with others without proper guidance, potentially resulting in serious consequences.

“I used to share metformin with my nephew, and he got severe diarrhea. After that, I never shared medications with anyone again.” (Vatcharavongvan and Puttawanchai, 2022)

“I use remaining medications first. I do not look at an expiratory date. [I look at] the date I received the medications and use the older ones first.” (Vatcharavongvan and Puttawanchai, 2022)

##### 3.4.1.1.8 Combing non-pharmacological treatment

In addition to medication treatment, healthcare providers also recommend assessing the patient’s condition and considering their preferences to incorporate non-pharmacological treatments, such as acupuncture, to improve symptoms.

“So, not even just medications based, but things like acupuncture, other kind of techniques that can be done. So, I think that something like pain is a very difficult problem to solve and we should be trying as many different things as we can.” (HCP) (Guilcher et al., 2020)

##### 3.4.1.2 Subtheme 2: health behavior strategies (6/320)

In health behavior strategies, patients should actively adjust their lifestyle to enhance the effectiveness of medication and maximize health benefits. Some patients need to be aware of the potential interactions between medications and food, adjusting their dietary habits to align with medication timing and mitigate risks. Regular exercise is also important for improving physical condition and mood, which can, in turn, enhance the effectiveness of medications. Additionally, maintaining a positive mindset and seeking psychological support can further strengthen patients’ management capabilities. Proper sleep management is essential to reduce stress and fatigue during the treatment process. Although these adjustments may cause inconvenience or impatience for patients, they are crucial for managing and improving health and supporting the overall effectiveness of medication. Healthcare providers should offer personalized recommendations based on the individual needs of each patient.

“Yeah, I keep moving, I keep doing you know, doing what I have to do. And going, going for a walk, I think that really helps. I’m sure, I’m absolutely certain exercise makes me feel better but yeah I, I, I feel quite tired.” (Cossart et al., 2022)

“... because we have to be at the pathology and then the clinic, you know by half past six in the morning, every single day for nearly a month it's like, you just spend your whole time trying to work yourself out. You’re really not mentally capable of taking in anymore.” (Cossart et al., 2022)

“You have to take it first thing in the morning, you have to stand up or at least sit upright, you cannot eat or drink for half an hour after you've done it, half an hour to an hour, and you have to be careful.” (Previdoli et al., 2024)

#### 3.4.2 Theme 2: support-oriented domains (185/320)

##### 3.4.2.1 Subtheme 1: process strategies (97/320)

In process strategies, patients need to develop proper belief of medication use, identify and address any abnormalities during the medication process, and optimize their medication strategies by acquiring accurate information. Healthcare providers should ensure that the medication plans they develop and implement align with the overall treatment goals and consider the patient’s needs for medication adjustment and optimization to achieve the best possible treatment outcomes.

##### 3.4.2.1.1 Developing proper medication belief

Many patients may adjust or even stop their medication on their own due to a lack of understanding of the medication’s mechanism, concerns about or experiences with side effects, not perceiving significant benefits, believing they have improved when symptoms lessen, resistance to long-term medication use, or due to the cost of medications. Additionally, some patients do not proactively monitor their health and rely entirely on healthcare providers, which can hinder effective treatment and potentially worsen their condition. This underscores the importance of the need for patients to establish proper medication belief.

“For a while, I thought I would just cut my pill in half because I live away from the pharmacist.” (Jallow et al., 2024)

“I’m worried now what have I. What’s the cause of this, you know? And is it that the medications need to be changed to accommodate what’s wrong with me?” (Foley et al., 2022)

“I do not use the furosemide anymore. I do not have any problems with urinating anymore. I did not ask the physician if I could stop using the medications.” (Dijkstra et al., 2022)

“If there is anything that is wrong. Then, they will contact you. At once. Therefore, I trust that.” (Holmqvist et al., 2019)

“If the injury is minor then it’s not totally affecting their daily life, they may only use the medication when things get a little worse. Even though they may be supposed to take it on a daily basis. If it’s not bothering them, they may not [take it]. Yeah, so the less severe the injury is, the more prone, they are to not adhering, essentially.” (HCP) (Guilcher et al., 2019)

##### 3.4.2.1.2 Acquiring accurate medication information

Healthcare providers, especially primary care providers, need further education to recognize reliable information sources and enhance their knowledge base through multidisciplinary collaboration. Utilizing community resources and strengthening coordination among healthcare providers is essential to offer patients accurate information. When providing education, it is important to consider the patient’s cultural background and needs, selecting appropriate methods of instruction. Additionally, helping patients gain more social support and encouraging family involvement in managing the patient’s treatment are crucial.

“I think my role as a nurse practitioner impacting the identification and management of polypharmacy in the geriatric population is really on a community level. I think my impact is really on providing the community, patients, students, and other disciplines [information] about Beers Criteria, aging and the toll on the body, and education.” (HCP) (Hernandez, 2017)

“I have to explain in detail. And I tell them, ‘The paper that you have from our hospital, that’s the one I’m going to be teaching you on.’ And then sometimes we get into this struggle with patients because they’ll say, ‘Well, I just saw my doctor yesterday or two weeks ago and he put me on medication [for fluid retention],’ which is a fluid pill. ‘He put me on that and now you guys tell me to stop it, so what do I do?’ I’m like, ‘Well, go by the most recent summary, which is what we - what I’m going over is they want you to stop it. But now, however, you need to see your primary care right away.’” (HCP) (Hannum et al., 2021)

“Maybe it’s the educational level of the person. If it’s really, really, rudimentary level then it’s a little bit - can be a little bit tough, you know, to reinforce certain ideas.” (HCP) (Guilcher et al., 2020)

“I think it would help to have, obviously more social support for these patients through case management and social work.” (HCP) (Hannum et al., 2021)

Some patients obtain medication information through various channels such as medication lists, package inserts, consultations with doctors or friends, and the internet. However, when being educated, patients may encounter information overload or incorrect information, leading to omissions or misunderstandings. Additionally, some patients do not actively seek information and make decisions on their own, or they may have misconceptions about the medication, which can negatively impact the safety and effectiveness of their treatment.

“I read about them. I just google them. Any time I get a new medicine, like headache medicine, I google it, and I find out what it does, what you can take with it or what you cannot take with it.” (Jallow et al., 2024)

“I prepared a medicine list, and I listed what I was concerned about health-wise for me [to ask the provider during visits].” (Jallow et al., 2024)

“Yes, some things one remembers, but it can be like stuffing too much information in, so to say. When you sit and go through a list like this, you know, and you concentrate, there may be something that gets lost, you know.” (Holmqvist et al., 2019)

“I do not ask no questions because I figure the doctor knows what he’s doing.” (Schöpf et al., 2018)

##### 3.4.2.1.3 Rational medication adjustment and optimization

Many patients wish to adjust their current medication regimens, seeking to control their condition with fewer doses or fewer types of medication. However, they have concerns about the potential risks of such adjustments and therefore desire comprehensive medication review and optimization. Some patients suggest using combination medications to reduce the complexity of management, but this approach may also pose challenges in monitoring and making necessary adjustments.

“I do sometimes wish I were not taking as many tablets, but while ever it's keeping me going, I'll take them. [laughs] Is the side effects better than, you know, what you're taking them for? That sort of thing goes through me head sometimes.” (Previdoli et al., 2024)

“Do you know when you’re years on a tablet too I think it’s, it was time to assess them. That’s my belief anyway. I was years going in there and it was the same ding dong, get a prescription, give him a prescription, and you take them and there were some of them now, some of them were bad anyway. I was too long on them anyway.” (Foley et al., 2022)

“Well perhaps, I’ve heard talk about maybe where you’re on two or three different tablets getting a tablet that contains, one tablet that contains the three drugs that you’re on. That would make matters easier for a lot of people, especially people that are on these drugs because they are long term so they’re not going to change very often. So that’s one way that would help.” (Williams et al., 2005)

“I do not honestly think so because there are different heart tablets and different cholesterol tablets and it is difficult enough to find the one that suits you rather than suddenly finding that they put two together and you cannot find one that suits you. If they were asking for a recommendation then I would say no.” (Williams et al., 2005)

Healthcare providers should align with the patient’s treatment goals, encouraging active participation in the decision-making process. They should comprehensively weigh the benefits and risks of medication adjustments and conduct dynamic medication reviews. Additionally, healthcare providers should enhance coordination and communication among themselves to optimize medication use, avoid inappropriate prescriptions, and ensure that patients receive the safest and most effective treatment plan.

“I think in the longer term it probably also, depends on the patients’ goals for themselves and whether they start valuing quality of care over quantity, or yeah, quality of life versus quantity of life, especially if their condition is starting to deteriorate. Sometimes they may not want to continue certain medications because of their long terms goals.” (HCP) (Guilcher et al., 2019)

“... in terms of I guess how inconvenient it is like if it’s something they were taking every four hours, you kind of have to stop whatever you’re doing to take the medication versus if you can give them something that’s long-acting that you only take twice a day.” (HCP) (Guilcher et al., 2019)

“Later on now in my career, I’ve taken on a different type of feeling about my approach. I understand that managing polypharmacy is an art as much as it is a science. You have to balance quality of life, risks and benefits, when prescribing medications to the older adult. I do not feel the need to fix everything.” (HCP) (Hernandez, 2017)

“It’s not cut and dry. I will identify patients with polypharmacy, but at the same time going through all of the medications and why they were put on the medication is such a web. You see that someone is on medications for legitimate issues, heart problems, high blood pressure, but then you have to step back and look at are we treating symptoms of other medications. Did you go to the urologist for incontinence because of the diuretic you were placed on for your blood pressure? It’s a scenario that gets repeated a lot.” (HCP) (Hernandez, 2017)

#### 3.4.2.2 Subtheme 2: resource strategies (88/320)

In resource strategies, patients need to actively exercise their initiative by expressing their needs and participating in medical decision-making to ensure that their preferences and requirements are fully considered. Additionally, patients should develop the ability to identify and effectively utilize social support networks, which can aid them in making more informed decisions during complex treatment processes. This approach ensures that their medication management is closely aligned with their personal treatment goals, ultimately leading to the best possible treatment outcomes.

##### 3.4.2.2.1 Exercising initiative

Most patients are able to exercise their initiative, actively participating in the treatment process to maintain a level of autonomy. Healthcare providers also emphasize the importance of patients having a proactive health mindset, strengthening their management capabilities through improved communication and involvement in decision-making. However, some patients tend to adopt a passive approach, being reluctant or unable to actively engage in the treatment process, thereby not fully realizing their self-management potential.

“Yes, as I was about to say. The responsibility must be mine almost. That I alert them if it would fail.” (Holmqvist et al., 2019)

“I have no problem, it's a very simple operation. I've never questioned with my GP as to whether it should change, I'm in the hands of the professionals.” (Previdoli et al., 2024)

“A lot of patients, particularly the older-old have a mentality of I just do whatever my provider tells me. They do not question the different specialties adding other medicines. It is up to me in primary care to be the gate keeper and inform the patient and their families.” (HCP) (Hernandez, 2017)

##### 3.4.2.2.2 Utilizing social support networks

Social support networks refer to the collection of social relationships and resources that individuals can rely on in their daily lives. In the context of medication management, patients can enhance their self-management capabilities by effectively utilizing these networks to gain emotional support, informational assistance, and practical help. However, some patients may face challenges when leveraging social support networks, such as concerns about burdening caregivers, not receiving thorough evaluation or accurate information support, and experiencing a lack of coordinated communication.

“Thankfully, I had my daughter. She came in every day. And she was in on all the conversations [about how to pack and take medicines].” (Cossart et al., 2022)

“When I have a problem, for example a side effect, then I go to the general practitioner.” (Dijkstra et al., 2022)

“I do not want my son to help me because I can do it myself. I do not want to disturb him.” (Vatcharavongvan and Puttawanchai, 2022)

“I asked the doctor on my recent visit ‘Aren’t you going to check my bones’ I said. However ‘No,’ she said, ‘they had not said anything from there [the hospital]’. You know, when you get old, they withdraw all such assessments.” (Holmqvist et al., 2019)

“I'm finding that difficult. It's between three lots, both consultants and the surgery and, yeah, and it's difficult for them because, you know, it's changing each time, and I phone the surgery and say, ‘I know my prescription needs to change because I was told that at the consultation’, and they say, ‘No, we have not got a letter from them, we cannot change it’.” (Previdoli et al., 2024)

Healthcare providers should assess patients’ social support networks, identifying existing resources and potential gaps, and provide targeted assistance to help patients build and strengthen these networks. This could involve establishing trust, collaborating with community resources, promoting multidisciplinary cooperation, enhancing coordination, and innovating support delivery methods. Additionally, by offering education and training, the capacity and knowledge of caregivers can be improved, ensuring they are able to effectively support the patient’s treatment process and ultimately enhance the patient’s treatment outcomes.

“... it can be you know, very overwhelming, I find. Particularly affecting sort of you know, sort of my clients who are males and they’re in their 30s, 40s even 50s who [prior to] their spinal cord injury had, you know, little to no interaction with the healthcare system. And now, you know have major healthcare needs. I find, you know for a lot of them, they kind of struggle in the beginning in terms of wrapping their head around it. I find for people who have been connected with the healthcare system longer, it’s sort of not as jarring.” (HCP) (Guilcher et al., 2019)

“Polypharmacy always gets sticky. I think mismanagement of pharmaceuticals is the main problem that most of my patients encounter. I think it takes a very skilled clinician to be able to piece everything together; piece all the specialists’ work together; piece all the transitions of care together.” (HCP) (Hernandez, 2017)

“And sometimes it’s important to address their non or informal caregivers on motivational speaking. The patient may be depressed, for example, or they may be unwilling to take their medication, in which case it is important for people in their life to help them stay motivated in order to adhere to the optimum medication therapy regimen.” (HCP) (Guilcher et al., 2019)

### 3.4.3 Theme 3: emotional and role management (58/320)

#### 3.4.3.1 Subtheme 1: activities strategies (24/320)

In activities strategies, patients need to integrate medications into their daily routines and use various methods to remind themselves to take their medications on time. Some patients may also need to handle complex packaging issues. Actively participating in meaningful activities is another key component of these strategies, which includes ensuring that treatment is not interrupted during outings, thereby allowing patients to manage their medication process more flexibly and effectively.

##### 3.4.3.1.1 Integrating medication into daily life

Most patients recognize the importance of taking their medication on time and use methods such as setting alarms, choosing strategic storage locations, checking pillbox status, relying on memory, and establishing fixed habits to remind themselves to take their medication. However, some patients, particularly younger ones, may forget to take their medication due to unexpected events like social gatherings, while elderly patients might forget due to memory decline. Additionally, some patients encounter difficulties in opening medication packaging, which can disrupt their medication process.

“You have to have a bit of a routine when you have so many things.” (Foley et al., 2022)

“I have a timer that I set every time I finish my meal. And when that timer dings, then I take my medication.” (Jallow et al., 2024)

“Well, it’s a lifestyle thing. I’m not always home for breakfast. I’m not always home for lunch. I’m not always home for dinner. I might want to go out and meet someone. Going out or I am sleeping in later than I normally would because I’ve been out the night before or, heaven forbid, I do not wake up at home. but you do not have your medication there. It’s one of those things, it’s lifestyle based, it does not fit in with a young person’s lifestyle.” (Tudball et al., 2015)

“I take them with my lunch. And when I do that I forget to take them entirely if I do not leave them on the table in the morning.” (Foley et al., 2022)

“I have problems with opening packages every day because of my arthrosis in both my hands. I found a way to handle this situation. I decided to prepare the intake for a whole week on one day a week. Then the pain is just once per week.” (Dijkstra et al., 2022)

##### 3.4.3.1.2 Planning for outings

Patients usually prepare their medications in advance once travel plans are confirmed, ensuring that their treatment is not interrupted during the trip. However, unexpected events such as sudden changes in itinerary, lack of access to luggage, insufficient medication supply, or time zone differences can cause difficulties with medication management, thereby disrupting the normal medication routine.

“So, if I go away on holidays [or a work trip], I have to think, ‘Do I have enough of my main medication to cover the period I’m going to be away? Will I be able to get them if I take my scripts, will I be able to get them where I’m going, or do I have to go and get more now? Do I have to go and get new scripts?’ I’ll take a couple of days extra, just in case there’s a change in plans.” (Tudball et al., 2015)

“I rarely miss medications, especially when I stay at home. The exception is when I go out; I sometimes forget to take medications with me.” (Vatcharavongvan and Puttawanchai, 2022)

“So a bit of a business, when I go to the Czech Republic, which I do every year, again visiting grandchildren and family, adjusting the time shift with the medicine is a bit of an issue. But I just work out what seems to be reasonable, stick somewhere in the middle.” (Tudball et al., 2015)

##### 3.4.3.2 Subtheme 2: internal strategies (31/320)

In internal strategies, patients should learn to identify and manage negative emotions related to medication use and maintain a positive mindset to minimize the impact of these emotions on their treatment. The complexity of managing multiple medications may cause psychological distress, but most patients cope with these challenges by focusing on the benefits of treatment and diverting their attention to other activities.

“You look at all the bottles up there and you just shake your head.” (Vandermause et al., 2016)

“If I am afraid of side effects or if I do not take medicines, I may have complications from diseases. I have to accept that fact. Take them. If there will be side effects, let them be. I am not worried at all.” (Vatcharavongvan and Puttawanchai, 2022)

“And suddenly it’s almost like you’ve regained your whole life, you can go out, you can go on holidays. You can go out for lunch with your friends and walk the length of the Shopping Centre. I mean it’s, it’s just amazing I’m telling you. Anybody who says it’s not a good thing, they’re not doing it right.” (Cossart et al., 2022)

“Yeah, I think it's always in the back of mind. I do not care, anybody who’s had a transplant must think that, and I do think that. But I do not dwell on it.” (Cossart et al., 2022)

##### 3.4.3.3 Subtheme 3: social interaction strategies (3/320)

In social interaction strategies, patients should actively maintain and develop social connections to restore their social functioning. Patients may choose to engage in selective social interactions, preferring to communicate with others who share the same condition, in order to gain encouragement and advice, thereby further facilitating the success of their treatment.

“If you're talking to somebody who knows exactly what you're talking about, it does make a difference.” (Cossart et al., 2022)

## 4 Discussion

This systematic review of 16 qualitative studies was to explore the process of medication self-management in polypharmacy from the perspectives of patients and healthcare providers, and to analyse the complex factors affecting medication self-management, which were mapped into the TEDSS framework (Cadel et al., 2020). The study found that while most patients could incorporate medication management into their routine self-care practices, there were still issues to be improved in medical management, support-oriented domains and emotion and role management.

Polypharmacy has become an increasingly common issue in modern healthcare, with a growing number of patients requiring long-term use of multiple medications to manage various conditions (Tang et al., 2022). As the number of medications increases, medication management becomes more complex, necessitating higher levels of medication literacy and personalized management strategies. However, a study by Plaza-Zamora et al. (Plaza-Zamora et al., 2020) found that only 34% of community pharmacy clients have adequate medication literacy. This finding aligns with the significant lack of rational medication knowledge among the elderly population (Mei et al., 2024). Additionally, research by Funk et al. (Funk et al., 2021) revealed that 76.7% of households store at least one medication improperly, consistent with our findings that many patients struggle to properly follow complex medication regimens. Due to insufficient medication information and beliefs, some patients even adjust or discontinue their medications on their own. These studies highlight the necessity of enhancing medication education and information provision in polypharmacy management to improve medication literacy, ensuring that patients not only understand the function of their medications and provide accurate medication history but also have the ability to access, correctly verify, store, and use them promptly, while effectively monitoring and managing the efficacy and side effects of the medications.

There is a close relationship between health behavior strategies and medication management. Research indicates that behaviors such as obesity and smoking are risk factors for polypharmacy (Piao et al., 2024). By adopting a healthy lifestyle, such as a balanced diet and regular exercise, patients can sometimes enhance the effectiveness of their medications and reduce their dependence on them (Gillies et al., 2007; Zhang et al., 2023; Koren et al., 2024). On the other hand, medication use may require patients to adjust their lifestyle to avoid potential side effects and adverse reactions. For instance, some medications might necessitate dietary adjustments to prevent drug-food interactions from affecting efficacy (Niederberger and Parnham, 2021; D'Alessandro et al., 2022). Therefore, healthcare providers should offer personalized guidance and ongoing support to help patients find the optimal balance between lifestyle adjustments and medication therapy. In this way, patients can not only manage their medications more effectively but also reduce the risk of adverse reactions and improve their overall health.

It is noteworthy that as modern healthcare systems increasingly shift responsibility onto patients and their social support networks, this shift, although intended to enhance patients’ self-management capabilities, may also increase their burden (May et al., 2014). Without adequate support and guidance, this burden could lead to poor management, resulting in suboptimal healthcare outcomes and an increase in healthcare service demand and costs. Consequently, polypharmacy management now requires a higher level of expertise from healthcare providers. To achieve minimally disruptive medicine, dynamically conducting medication reviews and optimization is particularly crucial (May et al., 2009). To this end, various tools need to be developed that assist healthcare providers (Fellenor et al., 2021; Urbańczyk et al., 2023) and patients (Dimitrow et al., 2023) in reviewing current medications, identifying those who might benefit from deprescribing interventions, and reducing the incidence of inappropriate prescriptions. This approach not only helps lower associated costs but also alleviates the daily burden on patients, thereby improving their quality of life.

A robust social support network can provide patients with essential emotional support, informational support, and practical assistance, thereby encouraging proactive health engagement and enhancing their self-management capabilities (Dhand et al., 2016). Many patients wish to adjust their current medication regimens, seeking to control their condition with fewer doses or fewer types of medication. Healthcare providers should assess patients’ social support networks and consider patients’ life needs, treatment goals, economic circumstances (Woodward et al., 2024), and personal preferences (Limenh et al., 2024) to offer personalized support and guidance to help patients build and strengthen their support systems. Additionally, healthcare providers should engage in education and training programs to improve their own health literacy, gain stronger medication knowledge, and enhance clinical judgment (Lüthold et al., 2024). By implementing effective resource allocation, promoting pharmacist integration, fostering multidisciplinary collaboration, and utilizing electronic medication verification tools (based on web or mobile applications), healthcare providers can improve coordination and deliver better services to patients (Ciudad-Gutiérrez et al., 2023; Naseralallah et al., 2023).

With the continuous advancement of technology, more and more innovative tools are being developed to help patients integrate medication management into their daily lives, effectively reducing the risk of forgetting medications (Kini and Ho, 2018). Such as smart pillboxes, medication reminder apps, and digital health monitoring systems. Travel is important for maintaining a positive mental state (Ybanez-Blomstrom et al., 2008), but it is crucial to develop a medication plan for patients during travel. This not only enhances their convenience and comfort but also strengthens their ability to handle emergencies. This includes strategies for managing time zone differences, insufficient medication supplies, unexpected trips, and missed doses, allowing patients to better manage their medication therapy while traveling and avoid health complications due to unexpected situations (Tudball et al., 2015). Additionally, focusing on psychological counseling is a key factor in improving patients’ quality of life. Teaching psychological counseling methods can help patients better manage their emotions when facing illness and treatment pressures, restore social functions, and improve overall health and life satisfaction (van Agteren et al., 2021).

## 5 Strengths and limitations

This study presents the first systematic review of the medication self-management process in polypharmacy from the attitudes and experiences of patients and healthcare providers, following a strict protocol and the ENTREQ guidelines to ensure a thorough, transparent, and repeatable review process. The study adopted the most common definition of polypharmacy, which facilitates the generalization and extension of the results. The studies included were evaluated using the CASP quality assessment checklist and were generally perceived to be of high quality, enhancing our reliance on their outcomes. In addition, we invited three healthcare providers and four patients to review the comprehensive themes, and their suggestions were incorporated into the determination of the final themes. This process not only helped to validate the research team’s analytical results, ensuring consistency with real-world situations, but also enriched our thematic analysis, making the results more comprehensive and targeted. The study helps in designing interventions that are more tailored to patient needs and supporting health policy making.

Despite its strengths, this systematic review has some significant limitations. The majority of the included studies are from high-income countries, revealing a gap in research from low and middle-income countries. Thus, interpreting these findings requires particular care, especially regarding low and middle-income countries. Additionally, this study only included English language articles, likely missing other relevant research that conforms to the criteria, and thus might have introduced some bias. The findings include insufficient details about emotional and role management, making it difficult to thoroughly analyse patients’ strategies in these areas. Finally, the professional backgrounds of the authors may have an impact on the conclusions of this review.

## 6 Conclusion

This study used the TEDSS model as a framework to analyse medication self-management process in polypharmacy from the perspectives of patients and healthcare providers and found that patients still have problems to improve in medical management, support-oriented domains and emotion and role management. Enhancing patients’ proactive health awareness, improving medication literacy, balancing lifestyle adjustments with medication therapy, dynamically reviewing and optimizing medications, strengthening patients’ social support networks, and helping patients integrate medication management into their daily life are the key elements that can effectively assist patients in self-managing their medications. Future research should focus on developing effective intervention strategies to further enhance self-management abilities. The insights gained from this study can help design specific interventions tailored to patients’ needs.

## Data Availability

The original contributions presented in the study are included in the article/Supplementary Material, further inquiries can be directed to the corresponding author.
