# Supplementary material for: Exploring medication self-management in polypharmacy: a qualitative systematic review of patients and healthcare providers perspectives
Source: Front Pharmacol. 2024 Sep 13;15:1426777. doi: 10.3389/fphar.2024.1426777 (PMC11456697; doi:10.3389/fphar.2024.1426777)
Supplement: Supplementary file 1 [file DataSheet3.docx]

**GRADE-CERQual Assessment Results**

| Review Finding | | Studies Contributing to the Review Finding | Assessment of Methodological Limitations | Assessment of Relevance | Assessment of Coherence | Assessment of Adequacy | Overall CERQual Assessment of Confidence |
| --- | --- | --- | --- | --- | --- | --- | --- |
| Theme1 medical management | | | | | | | |
| Finding1 | disease control strategies | Studies 1;2;5;6;8-13;15;16 | Minor limitations(6 studies with no or minor,3 studies with moderate and 3 studies with major) | Minor concerns | Minor concerns | Minor concerns | High |
| Finding2 | health behavior strategies | Studies 7;10;14 | Serious limitations(1 study with no or minor,1 study with moderate and 1 study with major ) | Moderate concerns | Minor concerns | Moderate concerns | Low |
| Theme2 support-oriented domains | | | | | | | |
| Finding3 | process strategies | Studies 1-16 | Minor limitations(7 studies with no or minor,5 studies with moderate and 4 studies with major) | Minor concerns | Minor concerns | Minor concerns | High |
| Finding4 | resources strategies | Studies 1;3;4;6-16 | Moderate limitations(5 studies with no or minor,5 studies with moderate and 4 studies with major) | Minor concerns | Minor concerns | Minor concerns | Moderate |
| Theme3 role and emotion management | | | | | | | |
| Finding5 | activities strategies | Studies 4;7-9;13;15;16 | Moderate limitations(3 studies with no or minor,3 studies with moderate and 1 study with major) | Moderate concerns | Minor concerns | Minor concerns | Moderate |
| Finding6 | internal strategies | Studies 1;2;4;5;7-9;11;12;14;15 | Minor limitations(5 studies with no or minor,4 studies with moderate and 2 studies with major) | Minor concerns | Minor concerns | Minor concerns | High |
| Finding7 | social interaction strategies | Study 7 | Minor limitations(1 study with no or minor) | Moderate concerns | Minor concerns | Serious concerns | Low |

**
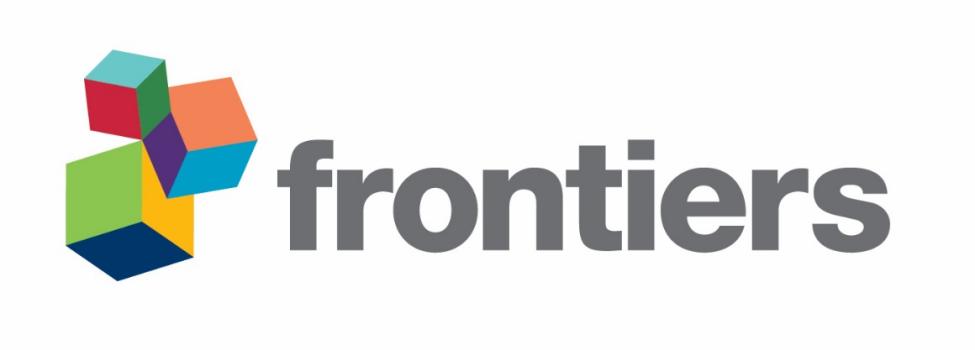
**
