# Supplementary material for: Exploring medication self-management in polypharmacy: a qualitative systematic review of patients and healthcare providers perspectives
Source: Front Pharmacol. 2024 Sep 13;15:1426777. doi: 10.3389/fphar.2024.1426777 (PMC11456697; doi:10.3389/fphar.2024.1426777)
Supplement: Supplementary file 2 [file DataSheet2.docx]

**Critical Appraisal Skills Program (CASP) Tool Results**

| Author/（year） | ① | ② | ③ | ④ | ⑤ | ⑥ | ⑦ | ⑧ | ⑨ | ⑩ | Overall Assessment |
| --- | --- | --- | --- | --- | --- | --- | --- | --- | --- | --- | --- |
| Holmqvist M.et al(2019) | Yes | Yes | Yes | Yes | Yes | No | Yes | Yes | Yes | Yes | No or minor |
| Fried TR.et al (2008) | Yes | Yes | Yes | Yes | Yes | No | Yes | Yes | Yes | Yes | No or minor |
| Andrea C.et al(2018) | Yes | Yes | Yes | Unclear | Unclear | No | Yes | Yes | Yes | Yes | Major |
| Jacqueline T.et al(2015) | Yes | Yes | Yes | Yes | Unclear | No | Yes | Yes | Yes | Yes | Moderate |
| Williams B et al(2005) | Yes | Yes | Yes | Yes | Yes | No | Yes | Yes | Yes | Yes | No or minor |
| Hannum, S. M. et al.（2021） | Yes | Yes | Yes | Yes | Yes | No | Yes | Yes | Yes | Yes | No or minor |
| Cossart, Amelia R et al（2022） | Yes | Yes | Yes | Unclear | Yes | Yes | Yes | Yes | Yes | Yes | No or minor |
| Previdoli, G.（2024） | Yes | Yes | Yes | Yes | Yes | No | No | Yes | Yes | Yes | Major |
| Foley, Louise(2022) | Yes | Yes | Yes | Unclear | Yes | No | Yes | Yes | Yes | Yes | Moderate |
| Guilcher, Sara J. T. et al（2019） | Yes | Yes | Unclear | Unclear | Unclear | No | Yes | Yes | Yes | Yes | Major |
| Vatcharavongvan, P.et al（2022） | Yes | Yes | Yes | Unclear | Yes | No | Yes | Unclear | Yes | Yes | Major |
| Hernandez, J.（2017） | Yes | Yes | Yes | Unclear | Yes | No | Yes | Yes | Yes | Yes | Moderate |
| Dijkstra, N. E.et al （2022） | Yes | Yes | Yes | Yes | Yes | No | Yes | Yes | Yes | Yes | No or minor |
| Vandermause, Roxanne et al（2016） | Yes | Yes | Yes | Unclear | Yes | No | Yes | Yes | Yes | Yes | Moderate |
| Guilcher, Sara J. T.et al（2020） | Yes | Yes | Yes | Yes | Yes | No | Yes | Yes | Yes | Yes | No or minor |
| Jallow F.et al(2024) | Yes | Yes | Yes | Yes | Unclear | No | Yes | Yes | Yes | Yes | Moderate |

Note ①Was there a clear statement of the aims of the research?;②Is a qualitative methodology appropriate?;③Was the research design appropriate to address the aims of the research?;④Was the recruitment strategy appropriate to the aims of the research?;⑤Was the data collected in a way that addressed the research issue?;⑥Has the relationship between researcher and participants been adequately considered?;⑦Have ethical issues been taken into consideration?;⑧Was the data analysis sufficiently rigorous?;⑨Is there a clear statement of findings?;⑩How valuable is the research?

**
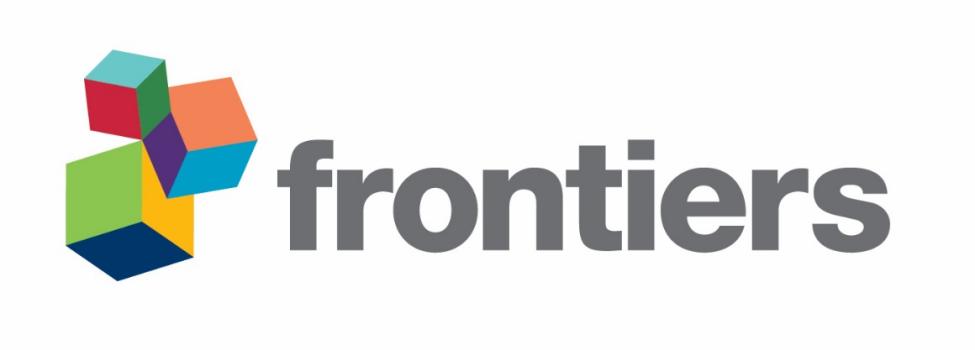
**
