# Supplementary material for: Exploring medication self-management in polypharmacy: a qualitative systematic review of patients and healthcare providers perspectives
Source: Front Pharmacol. 2024 Sep 13;15:1426777. doi: 10.3389/fphar.2024.1426777 (PMC11456697; doi:10.3389/fphar.2024.1426777)
Supplement: Supplementary file 3 [file DataSheet1.docx]

**Search strategy**

Date of Search: January 25, 2024 (updated August 6, 2024)

Number of results: **177**

| **PubMed** | | Total |
| --- | --- | --- |
| #1 | “Polypharmacy”[Mesh] | 6,851 |
| #2 | polypharmacy[Title/Abstract] OR polymedication[Title/Abstract] OR multiple medicine*[Title/Abstract] OR multiple medication*[Title/Abstract] OR multiple drug*[Title/Abstract] | 24,006 |
| #3 | #1 OR #2 | 26,316 |
| #4 | (("Self-Management"[Mesh]) OR "Self Administration"[Mesh]) OR "Self Care"[Mesh:NoExp] | 53,552 |
| #5 | self management[Title/Abstract] OR self-management[Title/Abstract] OR self government[Title/Abstract] OR self-government[Title/Abstract] OR self care[Title/Abstract] OR self-care[Title/Abstract] OR self monitor[Title/Abstract] OR self-monitor[Title/Abstract] OR self help[Title/Abstract] OR self-help[Title/Abstract] OR self medication[Title/Abstract] OR self-medication[Title/Abstract] OR self administration[Title/Abstract] OR self-administration[Title/Abstract] OR self maintenance[Title/Abstract] OR self-maintenance[Title/Abstract] OR self assessment[Title/Abstract] OR self-assessment[Title/Abstract] OR self control[Title/Abstract] OR self-control[Title/Abstract] OR self regulation[Title/Abstract] OR self-regulation[Title/Abstract] OR self nursing[Title/Abstract] OR self-nursing[Title/Abstract] | 109,015 |
| #6 | #4 OR #5 | 133,877 |
| #7 | "Qualitative Research"[Mesh] | 85,436 |
| #8 | qualitative research[Title/Abstract] OR qualitative[Title/Abstract] OR interview*[Title/Abstract] OR focus group[Title/Abstract] OR phenomenology[Title/Abstract] OR grounded theory[Title/Abstract] OR ethnography*[Title/Abstract] OR content analysis[Title/Abstract] OR thematic analysis[Title/Abstract] OR phenomenological[Title/Abstract] OR experience*[Title/Abstract] OR narrative research[Title/Abstract] OR expectation*[Title/Abstract] OR perception*[Title/Abstract] OR view*[Title/Abstract] OR attitude*[Title/Abstract] OR feeling*[Title/Abstract] | 2,877,756 |
| #9 | #7 OR #8 | 2,882,079 |
| #10 | #3 AND #6 AND #9 | **166** |

Date of Search: January 25, 2024 (updated August 6, 2024)

Number of results: **3052**

| **Web of science** | | Total |
| --- | --- | --- |
| #1 | TS=(polypharmacy or polymedication or multiple medicine* or multiple medication* or multiple drug*) | 194,743 |
| #2 | TS=(self management OR self-management OR self government OR self-government OR self care OR self-care OR self monitor OR self-monitor OR self help OR self-help OR self medication OR self-medication OR self administration OR self-administration OR self maintenance OR self-maintenance OR self assessment OR self-assessment OR self control OR self-control OR self regulation OR self-regulation OR self nursing OR self-nursing) | 567,553 |
| #3 | TS=(qualitative research OR qualitative OR interview* OR focus group OR phenomenology OR grounded theory OR ethnography* OR content analysis OR thematic analysis OR phenomenological OR experience* OR narrative research OR expectation* OR perception* OR view* OR attitude* OR feeling*) | 3,560,531 |
| #4 | #1 AND #2 AND #3 | **2841** |

Date of Search: January 25, 2024 (updated August 6, 2024)

Number of results: **1693**

| **Cochrane Library** | | Total |
| --- | --- | --- |
| #1 | MeSH descriptor: [Polypharmacy] explode all trees | 412 |
| #2 | (polypharmacy or polymedication or multiple medicine* or multiple medication* or multiple drug*):ti,ab,kw | 56,715 |
| #3 | #1 OR #2 | 56,715 |
| #4 | MeSH descriptor: [Self-Management] explode all trees | 1,042 |
| #5 | MeSH descriptor: [Self Administration] explode all trees | 851 |
| #6 | MeSH descriptor: [Self Care] this term only | 5,452 |
| #7 | (self management OR self-management OR self government OR self-government OR self care OR self-care OR self monitor OR self-monitor OR self help OR self-help OR self medication OR self-medication OR self administration OR self-administration OR self maintenance OR self-maintenance OR self assessment OR self-assessment OR self control OR self-control OR self regulation OR self-regulation OR self nursing OR self-nursing):ti,ab,kw | 132,702 |
| #8 | #4 OR #5 OR #6 OR #7 | 132,702 |
| #9 | MeSH descriptor: [Qualitative Research] explode all trees | 2095 |
| #10 | (qualitative research OR qualitative OR interview* OR focus group OR phenomenology OR grounded theory OR ethnography* OR content analysis OR thematic analysis OR phenomenological OR experience* OR narrative research OR expectation* OR perception* OR view* OR attitude* OR feeling*):ti,ab,kw | 296,714 |
| #11 | #9 OR #10 | 296,714 |
| #12 | #3 AND #8 AND #11 | **1600** |

Date of Search: January 25, 2024 (updated August 6, 2024)

Number of results: **307**

| **Embase** | | Total |
| --- | --- | --- |
| #1 | 'polypharmacy'/exp | 24,805 |
| #2 | polypharmacy:ab,ti OR polymedication:ab,ti OR 'multiple medicine*':ab,ti OR 'multiple medication*':ab,ti OR 'multiple drug*':ab,ti | 36,208 |
| #3 | #1 OR #2 | 46,007 |
| #4 | 'self care'/exp | 106,094 |
| #5 | 'drug self administration'/exp | 13,422 |
| #6 | 'self management':ab,ti OR 'self government':ab,ti OR 'self care':ab,ti OR 'self monitor':ab,ti OR 'self help':ab,ti OR 'self medication':ab,ti OR 'self administration':ab,ti OR 'self maintenance':ab,ti OR 'self assessment':ab,ti OR 'self control':ab,ti OR 'self regulation':ab,ti OR 'self nursing':ab,ti | 143,998 |
| #7 | #4 OR #5 OR #6 | 191,415 |
| #8 | 'qualitative research'/exp | 123,215 |
| #9 | 'qualitative research':ab,ti OR qualitative:ab,ti OR interview*:ab,ti OR 'focus group':ab,ti OR phenomenology:ab,ti OR 'grounded theory':ab,ti OR ethnography*:ab,ti OR 'content analysis':ab,ti OR 'thematic analysis':ab,ti OR phenomenological:ab,ti OR experience*:ab,ti OR 'narrative research':ab,ti OR expectation*:ab,ti OR perception*:ab,ti OR view*:ab,ti OR attitude*:ab,ti OR feeling*:ab,ti | 3,815,203 |
| #10 | #9 OR #10 | 3,823,103 |
| #11 | #3 AND #7 AND #10 | **292** |

Date of Search: January 25, 2024 (updated August 6, 2024)

Number of results: **169**

| **CINAHL** | | Total |
| --- | --- | --- |
| #1 | MH polypharmacy OR TI ( polypharmacy or polymedication or multiple medicine* or multiple medication* or multiple drug* ) OR AB ( polypharmacy or polymedication or multiple medicine* or multiple medication* or multiple drug* ) | 14,018 |
| #2 | MH ( self management OR self administration OR self-care ) OR TI ( self management OR self-management OR self government OR self-government OR self care OR self-care OR self monitor OR self-monitor OR self help OR self-help OR self medication OR self-medication OR self administration OR self-administration OR self maintenance OR self-maintenance OR self assessment OR self-assessment OR self control OR self-control OR self regulation OR self-regulation OR self nursing OR self-nursing ) OR AB ( self management OR self-management OR self government OR self-government OR self care OR self-care OR self monitor OR self-monitor OR self help OR self-help OR self medication OR self-medication OR self administration OR self-administration OR self maintenance OR self-maintenance OR self assessment OR self-assessment OR self control OR self-control OR self regulation OR self-regulation OR self nursing OR self-nursing ) | 91,192 |
| #3 | MH qualitative research OR TI ( qualitative research OR qualitative OR interview* OR focus group OR phenomenology OR grounded theory OR ethnography* OR content analysis OR thematic analysis OR phenomenological OR experience* OR narrative research OR expectation* OR perception* OR view* OR attitude* OR feeling* ) OR AB ( qualitative research OR qualitative OR interview* OR focus group OR phenomenology OR grounded theory OR ethnography* OR content analysis OR thematic analysis OR phenomenological OR experience* OR narrative research OR expectation* OR perception* OR view* OR attitude* OR feeling* ) | 1,081,817 |
| #4 | #1 AND #2 AND #3 | **167** |

Date of Search: January 25, 2024 (updated August 6, 2024)

Number of results: **104**

| **PsycINFO** | | Total |
| --- | --- | --- |
| #1 | MA polypharmacy OR TI ( polypharmacy or polymedication or multiple medicine* or multiple medication* or multiple drug* ) OR AB ( polypharmacy or polymedication or multiple medicine* or multiple medication* or multiple drug* ) | 6,424 |
| #2 | MA ( self management OR self administration OR self-care ) OR TI ( self management OR self-management OR self government OR self-government OR self care OR self-care OR self monitor OR self-monitor OR self help OR self-help OR self medication OR self-medication OR self administration OR self-administration OR self maintenance OR self-maintenance OR self assessment OR self-assessment OR self control OR self-control OR self regulation OR self-regulation OR self nursing OR self-nursing ) OR AB ( self management OR self-management OR self government OR self-government OR self care OR self-care OR self monitor OR self-monitor OR self help OR self-help OR self medication OR self-medication OR self administration OR self-administration OR self maintenance OR self-maintenance OR self assessment OR self-assessment OR self control OR self-control OR self regulation OR self-regulation OR self nursing OR self-nursing ) | 127,583 |
| #3 | MA qualitative research OR TI ( qualitative research OR qualitative OR interview* OR focus group OR phenomenology OR grounded theory OR ethnography* OR content analysis OR thematic analysis OR phenomenological OR experience* OR narrative research OR expectation* OR perception* OR view* OR attitude* OR feeling* ) OR AB ( qualitative research OR qualitative OR interview* OR focus group OR phenomenology OR grounded theory OR ethnography* OR content analysis OR thematic analysis OR phenomenological OR experience* OR narrative research OR expectation* OR perception* OR view* OR attitude* OR feeling* ) | 1,834,611 |
| #4 | #1 AND #2 AND #3 | **101** |

Date of Search: January 25, 2024 (updated August 6, 2024)

Number of results: **305**

| **MEDLINE** | | Total |
| --- | --- | --- |
| #1 | MH polypharmacy OR TI ( polypharmacy or polymedication or multiple medicine* or multiple medication* or multiple drug* ) OR AB ( polypharmacy or polymedication or multiple medicine* or multiple medication* or multiple drug* ) | 44,109 |
| #2 | MH ( self management OR self administration OR self-care ) OR TI ( self management OR self-management OR self government OR self-government OR self care OR self-care OR self monitor OR self-monitor OR self help OR self-help OR self medication OR self-medication OR self administration OR self-administration OR self maintenance OR self-maintenance OR self assessment OR self-assessment OR self control OR self-control OR self regulation OR self-regulation OR self nursing OR self-nursing ) OR AB ( self management OR self-management OR self government OR self-government OR self care OR self-care OR self monitor OR self-monitor OR self help OR self-help OR self medication OR self-medication OR self administration OR self-administration OR self maintenance OR self-maintenance OR self assessment OR self-assessment OR self control OR self-control OR self regulation OR self-regulation OR self nursing OR self-nursing ) | 167,873 |
| #3 | MH qualitative research OR TI ( qualitative research OR qualitative OR interview* OR focus group OR phenomenology OR grounded theory OR ethnography* OR content analysis OR thematic analysis OR phenomenological OR experience* OR narrative research OR expectation* OR perception* OR view* OR attitude* OR feeling* ) OR AB ( qualitative research OR qualitative OR interview* OR focus group OR phenomenology OR grounded theory OR ethnography* OR content analysis OR thematic analysis OR phenomenological OR experience* OR narrative research OR expectation* OR perception* OR view* OR attitude* OR feeling* ) | 2,879,444 |
| #4 | #1 AND #2 AND #3 | **284** |

**
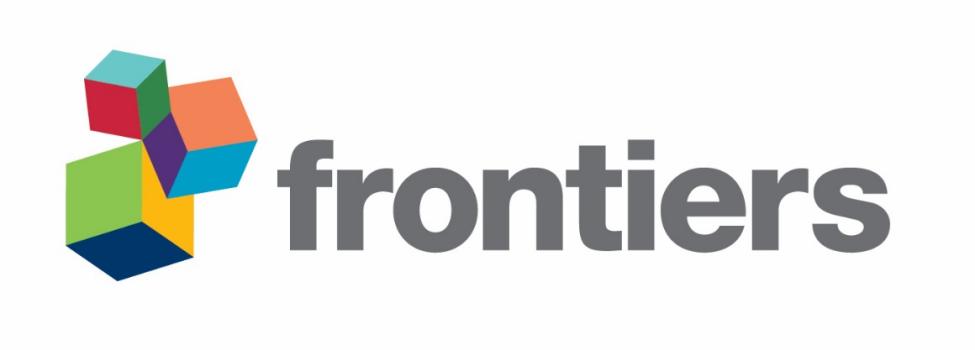
**
